# Effect of moderate hypothermic circulatory arrest on neurological outcomes in elderly patients undergoing replacement of the thoracic aorta

**DOI:** 10.1186/s43044-020-00043-7

**Published:** 2020-03-30

**Authors:** Mohamed Salem, Christine Friedrich, Alexander Thiem, Mostafa Ahmed Salem, Thomas Puehler, Rene Rusch, Rouven Berndt, Jochen Cremer, Assad Haneya

**Affiliations:** Department of Cardiovascular Surgery, University Hospital of Schleswig-Holstein, Campus Kiel, Arnold-Heller-Straße 3, 24105 Kiel, Germany

**Keywords:** Stroke in cardiac patient, Ascending aorta, Hypothermic circulatory arrest

## Abstract

**Background:**

Various studies evaluated the relationship between hypothermic circulatory arrest and neurological outcome in patients undergoing replacement of ascending aorta. The current analysis focuses on the effect of moderate hypothermic circulatory arrest (MHCA) on elderly patients. The aim of our study was to evaluate the impact of MHCA on neurological outcomes in elderly patients undergoing replacement of the ascending aorta.

**Results:**

We retrospectively analyzed 905 consecutive patients, who underwent elective replacement of ascending aorta in MHCA (24 ± 2 °C, nasopharyngeal) between 2001 and 2015. Patients with acute aortic dissection were excluded from this study. Patients were divided into two groups: those aged 75 years and older (elderly group 22.4%, *n* = 203) and those younger than 75 years (younger group 77.6%, *n* = 702).

The average age was 63.2 ± 10.2 in the young group vs. 78.7 ± 3.0 years in elderly group (*p* < 0.001). The elderly group had a significantly higher EuroSCORE II [26.7% (18.1, 36.3) vs. 11.6% (7.4, 19.9); *p* < 0.001)]. The incidence of coronary heart disease (49.8% vs. 35.6%, *p* < 0.001) and chronic renal failure (17.2% vs. 9.1%, *p* = 0.001) was significantly higher in the elderly group. Intraoperatively, the time of MHCA [14 min (12, 17) vs. 15 min (12, 18); *p* = 0.42], cardiopulmonary bypass [139 min (110, 183) vs. 144 min (113, 189); *p* = 0.225], and cross-clamping [91 min (63, 116) vs. 92 min (65, 127); *p* = 0.348] was similar in both groups. Postoperatively, a higher incidence of delirium was significantly reported in the elderly group (24.1% vs. 9.0%, *p* < 0.001). However, there was no significant difference regarding neurological complications between both groups. A 30-day mortality was acceptable for the elderly group, but significantly higher compared with the younger group (7.1% vs. 3.5%, *p* = 0.031).

**Conclusions:**

Our study suggests that surgical replacement of the ascending aorta in MHCA can also be applied safely in elderly patients without increasing the risk of severe neurological complications.

## Background

Replacement of the ascending aorta due to calcification and aneurysm is a major concern in cardiovascular surgery and is associated with a high risk of neurological complications [1]. Cerebral protection during this procedure is considered a major concern in successful thoracic aortic surgery due to its direct impact on the postoperative prognosis [2]. Beside antegrade and retrograde cerebral perfusion, MHCA is considered as one of the most preferred and successful methods to lower oxygen consumption and avoid brain tissue injury during the surgical replacement of the ascending aorta.

Various studies were carried out to analyze the effect of each method on postoperative neurological outcome. However, there is still a huge debate between cardiovascular surgeons regarding the best method of protecting the cerebral tissue during the duration of circulatory arrest.

The hypothermic circulatory arrest is considered the established technique for cerebral tissue protection during the surgical procedure of the ascending aorta and aortic arch due to its easy and safe application compared with antegrade and retrograde cerebral perfusion which is associated with various limitations [3].

Due to the marked increase in life expectancy, the number of patients over 75 years who undergo such a kind of operations also increases due to various medical and political considerations [4]. Various studies were done to figure out the relationship between the hypothermic circulatory arrest and the postoperative neurological outcome in general. In our analysis, we focused on the effect of MHCA on elderly patients more than 75 years old undergoing replacement of ascending aorta in comparison to younger patients regarding the postoperative neurological outcome.

In the current analysis, we studied the effect of MHCA during the replacement of the ascending aorta on the elderly patients.

## Methods

### Patient population

**Fig. 1:**
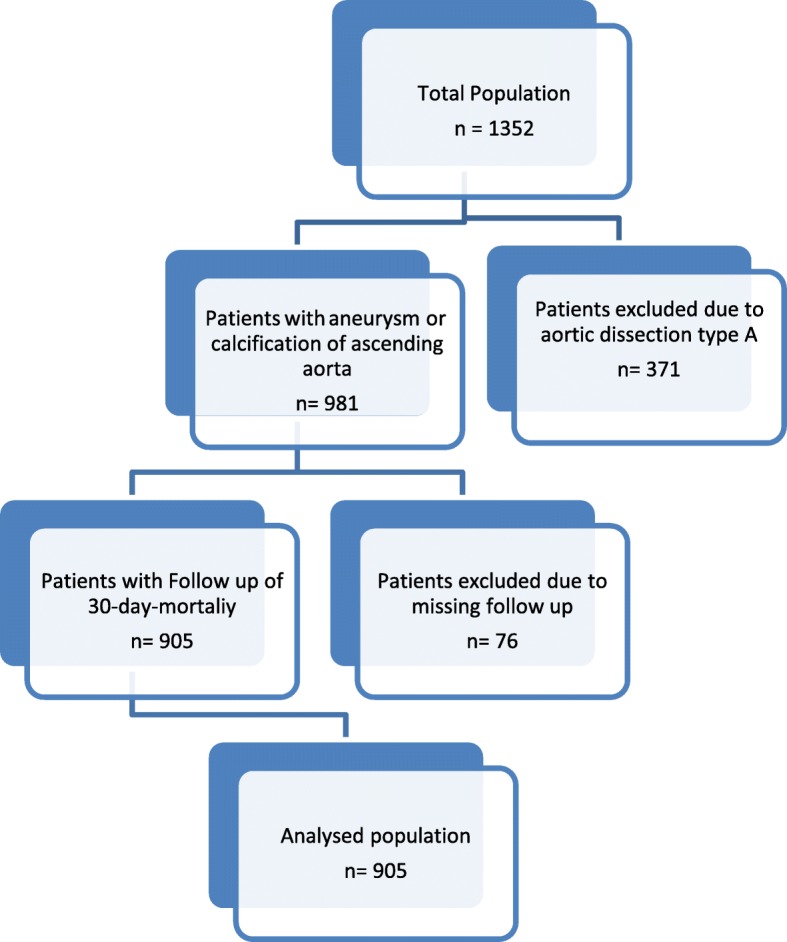
Consort diagram of the total population with exclusion

This is a retrospective study, enclosing 905 consecutive patients who underwent replacement of the ascending aorta due to calcification or aneurysm using MHCA (24 ± 2 °C, nasopharyngeal) in our center till 2015, either isolated or combined with other procedures (coronary artery bypass grafting, valve replacement, etc.). Patients with aortic dissection, as well as patients with redo operations and missed follow-up, were excluded from the study (Fig. 1).

Patients were divided into two groups: those aged 75 years and older (elderly group, 22.4%) and those younger than 75 years (younger group, 77.6%). The limit whether older or younger than 75 years old was settled according to a study from Friedrich et al. (2007), which in collaboration with the German Federal Quality Assurance Office (Bundesgeschäftsstelle Qualitätssicherung, BQS) analyzed the risk profiles of elderly patients by means of data sets from all cardiac surgical centers in Germany for the year 2007. The results showed that those patients over age 75 had significantly more prognosis-determining comorbidities and risk factors with higher complication rates and mortality compared to patients younger in age (e.g., a 4.3-fold risk elevation for renal failure, a 3.0-fold elevation for neurological adverse outcomes, and 3.7-fold elevation for in-hospital mortality) [4].

The primary endpoint was the postoperative neurologic complications. Secondary endpoints were a 30-day mortality and postoperative course of various body organs (e.g., ventilation time, bleeding, acute renal failure).

Data were collected and extracted from the institution’s database and from medical records. The study protocol was approved by the local Ethics Committee in Kiel, Germany (D417/17), and the necessary individual patient consent was obtained during the hospital stay.

### Patient management

Calcification or aneurysm of the ascending aorta was diagnosed either during routine X-ray investigation showing severe calcification of ascending aorta, routine echocardiographic assessment, or during coronary angiography prior to other cardiac surgery. The aortic calcification was also diagnosed and detected intraoperatively during other elective cardiac surgery such as CABG or aortic valve replacement within the direct palpation of the aorta. The decision to replace the ascending aorta was taken due to the inability of cross-clamping of the aorta due to the extensive calcification. When calcification of the ascending aorta was suspected preoperatively, a non-contrast computed tomography (CT-scan) was performed to present the exact location and extension of calcification. All patients were questioned at hospital admission for any history of neurological events as stroke or transient ischemic attack (TIA) as well as the presence of any medical records from the neurologist, computed tomography or magnetic resonance imaging. Patients were well investigated for neurological symptoms and signs, and any findings were documented on the admission sheet for further use. Without any exclusion, all patients were investigated for carotid arteries stenosis through carotid Doppler sonography. If there is a stenosis over 50–60%, a CT carotid artery angiography was carried out preoperatively and a vascular surgical consultant was contacted, to prove the indication of further surgical intervention.

One of the strict criteria in the data assembly postoperatively was that the neurological adverse outcome should be of new onset after the surgical procedure and not documented in the preoperative admission sheet.

The monitoring of tissue oxygenation of cerebrum intraoperatively was carried out by near-infrared spectroscopy (NIRS). Postoperative neurological sensory or motor deficits, if presented, were consulted directly by a neurologist and categorized according to a well-established neurological assessment. The results of the assessment were documented in patients’ file from the attending neurologist, followed by the head and neck computer tomography as well as, in many cases, CT angiography for the carotid arteries to estimate the extent of stroke and brain ischemia. MRI was required in some cases from the neurologists. After CT/MRI, a neurologist was consulted again for further plans. Delirium was measured by the Confusion Assessment Method (CAM) (https://www.icudelirium.org/medical-professionals/delirium/monitoring-delirium-in-the-icu) and was performed routinely per each shift. CAM was performed when the Richmond-Scale was more than − 4. Slightly known degree of delirium was mostly resolved rapidly under saline infusion and antipsychiatry drugs. In case of sever delirium, which requires patient fixation, a psychiatrist was consulted.

### Surgical procedure

All operations were performed by senior surgeons. A standard median sternotomy followed by longitudinal pericardiotomy was carried out under general anesthesia. Direct cannulation of the distal ascending aorta was used for arterial cannulation in most cases. In cases of severe calcification of ascending aorta till its arch and inability of its distal cannulation, we used the transatrial cannulation of the left ventricle via the right upper pulmonary vein as an alternative [5]. Venous drainage was performed through cannulation of the right atrium with a common two-stage venous cannula. A standard antegrade and retrograde injection of cold blood cardioplegic solution for myocardial protection was performed in all cases. The cardiopulmonary bypass (CPB) was conducted with MHCA with core temperature between 24 ± 2 °C which was measured nasopharyngeal. Brain tissue oxygenation was monitored by near infrared spectroscopy (NIRS). After suturing of the distal anastomosis, residual air was removed by restarting retrograde perfusion via the venous cannula and followed by slow antegrade perfusion. Continuous CO_2_ insufflation was used as a standard for the cardiac de-airing. After insertion of the perfusion cannula directly in the vascular graft, CPB restarted again. After conducting the proximal anastomosis, a cardiac de-airing is carried out before opening the clamped aortic prosthesis. Transoesophageal echocardiography was performed to control the presence of residual air in the left side of the heart. During rewarming, other procedures such as CABG or valve replacement were implemented if required.

### Statistical analysis

Statistical analysis was performed using the SPSS 18.0 software (SPSS, Chicago, IL, USA). Normality of continuous variables was assessed by Kolmogorow-Smirnow test. Values of continuous data are presented as mean ± standard deviation or as median with range or interquartile range when appropriate and compared by unpaired *t* test, whereas not normally distributed continuous were compared by Mann-Whitney *U* test. Categorical variables are displayed as frequency distributions (*n*) and simple percentages (%). Univariate comparison between the groups for categorical variables was made using the chi^2^ test and the Fisher’s exact test when appropriate. Statistical significance was considered when *p* ≤ 0.05. Logistic regression analysis was used to determine the hazards ratio (HR) of risk factors upon the 30-day survival time through backward selection with Likelihood ratio. Variables included in the regression analysis were age, EuroSCORE II, aortic aneurysm, aortic calcification, COPD, coronary heart disease, chronic renal insufficiency, additional CABG, and cardiopulmonary bypass time.

## Results

The average age was 63.2 ± 10.2 years in the young group vs.78.7 ± 3.0 years in the elderly group (*p* < 0.001). Female gender was more frequent in the elderly group (39.4% vs. 31.5%, *p* = 0.035). Accordingly, the elderly group had a significantly higher EuroSCORE II than the younger group [26.7% (18.1, 36.3) vs. 11.6% (7.4, 19.9); *p* = < 0.001)]. Coronary heart disease was significantly more frequent in the elderly group (49.8% vs. 35.6%, *p* < 0.001) with more atrial fibrillation (23.6% vs. 16.4%, *p* = 0.018). Chronic renal failure was significantly more often in elderly group (17.2% vs. 9.1%, *p* = 0.001) as well as type 2 diabetes mellitus (16.3% vs. 10.7%, *p* = 0.031). Patients who suffered from an aortic aneurysm represented 86.7% of the elderly group vs. 92.2% of the younger group (*p* = 0.017). Other risk factors showed no significant difference between both groups (Table 1).

**Table 1:** Demographic data, preoperative risk factors

	Total	Young group 702 (77.6%)	Elderly group 203 (22.4%)	*p* value
Mean age, years	66.7 ± 11.1 69.2 (61.0, 74.4)	63.2 ± 10.2 66.3 (57.5, 70.9)	78.7 ± 3.0 78.0 (76.3, 80.1)	**< 0.001**
Female gender, *n*	301 (33.3%)	221 (31.5%)	80 (39.4%)	**0.035**
Body mass index, kg/m^2^	26.5 (24.2, 29.4)	26.8 (24.2, 29.7)	25.4 (23.7, 27.8)	**< 0.001**
EuroSCORE II%	4.12 (2.38, 7.13)	11.6% (7.4, 19.9)	26.7% (18.1, 36.3)	**< 0.001**
Ejection fraction, %	64 (53, 70)	62 (51, 70)	65 (55, 70)	0**.**622
Aortic aneurysm, *n*	823 (90.9%)	647 (92.2%)	176 (86.7%)	**0.017**
Diameter of aneurysm, mm	52 (50, 57)	52 (50, 56)	52 (49, 59)	0.574
Aortic calcification, *n*	113 (12.5%)	55 (7.8%)	27 (13.3%)	0.017
Acute myocardial infarction, *n*	10 (1.1%)	8 (1.1%)	2 (1.0%)	1.000
Diabetes mellitus type II, *n*	108 (11.9%)	75 (10.7%)	33 (16.3%)	**0.031**
Peripheral vascular disease, *n*	108 (11.9%)	34 (4.8%)	17 (8.4%)	0.055
Arterial hypertension, *n*	687 (75.9%)	523 (74.5%)	164 (80.8%)	0.065
Atrial fibrillation, *n*	163 (18.0%)	115 (16.4%)	48 (23.6%)	**0.018**
Chronic obstructive pulmonary disease, *n*	104 (11.5%)	74 (10.6%)	30 (14.8%)	0.097
Coronary heart disease, *n*	350 (38.8%)	249 (35.6%)	101 (49.8%)	**< 0.001**
Chronic renal failure, *n*	99 (11.0%)	64 (9.1%)	35 (17.2%)	**0.001**
Renal replacement therapy, *n*	10 (1.1%)	8 (1.1%)	2 (1.0%)	**1.000**

The intraoperative data analysis revealed that the isolated replacement of supra-coronary ascending aorta was performed in 63.1% of the elderly group and 51.1% of the younger group. Aortic valve replacement due to either aortic valve insufficiency or stenosis was carried out in 52.2% in elderly patients vs. 50.7% in younger patients, while CABG was performed more often in the elderly (28.6% vs. 23.4%), however not reaching statistical significance. Partial replacement of the aortic arch was performed in 22.6% of elder patients vs. 21.7% in young patients. The CPB time [144 min (113, 189) vs. 139 min (110, 183); *p* = 0.225] as well as the cross-clamping time [92 min (65, 127) vs. 91 min (63, 116); *p* = 0.348] showed no significant difference between old and young patients. Time of circulatory arrest was nearly the same in both groups [15 min (12, 18) vs.14 min (12, 17); *p* = 0.42]. Direct cannulation of the ascending aorta (85.6% in elderly group vs. 78.6% in young group) was the most performed cannulation strategy (Table 2).

**Table 2:** Intraoperative course

	Total	Young group	Elderly group	*p* value
Surgical procedure				
Interposition graft alone, *n*	508 (56.1%)	380 (51.1%)	128 (63.1%)	0.024
Interposition graft with partial arch replacement, *n*	203 (22.4%)	159 (22.6%)	44 (21.7%)	0.769
Interposition graft with total arch replacement, *n*	29 (3.2%)	21 (3.0%)	8 (3.9%)	0.501
Interposition graft with separated aortic valve replacement, *n*	462 (51.0%)	356 (50.7%)	106 (52.2%)	0.706
Composite graft replacement of aortic root with reimplantation of coronary arteries, *n*	149 (16.5%)	124 (17.7%)	25 (12.3%)	0.070
Valve sparing aortic root replacement, *n*	63 (7.0%)	56 (8.0%)	7 (3.4%)	**0.026**
Performed CABG, *n*	222 (24.6%)	164 (23.4%)	58 (28.6%)	0.131
Length of surgery, min	250 (203, 308)	252 (205, 313)	240 (200, 294)	0.187
Cardiopulmonary bypass time, min	142 (113, 187)	144 (113, 189)	139 (110, 183)	0.225
Cross-clamp time, min	92 (65, 125)	92 (65, 127)	91 (63, 116)	0.348
Circulatory arrest, min	14 (12, 18)	14 (12, 17)	15 (12, 18)	0.419
Temperature, °C	24 ± 2	24 ± 2	24 ± 2	1.0
Number of packed red blood cells, units	2 (0, 4)	2 (0, 4)	2 (1, 4)	**0.002**
Number of platelets concentrate, units	1 (0, 1)	1 (0, 2)	1 (0, 1)	0.753
Arterial cannulation, *n*				
Ascending aorta	680 (80.2%)	520 (78.7%)	160 (85.6%)	
Aortic arch	107 (12.6%)	93 (14.1%)	14 (7.5%)	
Transatrial through the left ventricle	48 (5.7%)	36 (5.4%)	12 (6.4%)	
Femoral artery	10 (1.2%)	9 (1.4%)	1 (0.5%)	

Postoperative data analysis showed no significant difference in inotrope requirement or amount of blood loss during admission in the intensive care unit. The incidence of re-exploration due to bleeding or cardiac tamponade was unexpectedly lower in the elderly group than in the younger group (3.9% vs. 7.3%, *p* = 0.090). The incidence regarding the postoperative cardiac arrhythmia, temporary renal dialysis, prolonged mechanical ventilation, and deep wound infection were significantly higher in the elderly group than in the younger group. The overall hospital stays showed significantly differences between both groups. In our analysis, the elderly group suffered significantly more often from postoperative delirium than the younger group (24.1% vs. 9.0%, *p* = < 0.001). However, postoperative stroke showed no significant difference (4.9% vs. 6.0%, *p* = 0.569). A 30-day mortality was satisfactory for the elderly group, but significantly higher compared to the younger group (7.1% vs. 3.5%, *p* = 0.031) (Table 3).

**Table 3:** Postoperative data and outcomes

	Total	Young group	Elderly group	*p* value
Postoperative status				
Stable	325 (36.0%)	258 (36.8%)	67 (33.3%)	
Stable with low-dose catecholamines	533 (59.1%)	411 (58.6%)	122 (60.7%)	
Stable with high-dose catecholamines	33 (3.7%)	22 (3.1%)	11 (5.5%)	
IABP/ECLS with catecholamines	10 (1.1%)	9 (1.3%)	1 (0.5%)	
48-h drainage loss, mL	600 (400, 950)	550 (358, 900)	600 (400, 1150)	**0.016**
Re-exploration for bleeding, *n*	59 (6.5%)	51 (7.3%)	8 (3.9%)	0.090
Ventilation time, h	17 (12 31)	17 (11, 26)	20 (15, 57)	**< 0.001**
Tracheotomy, *n*	69 (7.6%)	50 (7.1%)	19 (9.4%)	0.293
Pulmonary infection	58 (6.4%)	37 (5.3%)	21 (10.3%)	**0.009**
New onset of hemodialysis, *n*	48 (5.3%)	36 (5.1%)	12 (5.9%)	0.667
Postoperative atrial fibrillation, *n*	140 (15.7%)	95 (13.7%)	45 (22.6%)	**0.002**
Postoperative delirium, *n*	112 (12.4%)	63 (9.0%)	49 (24.1%)	**< 0.001**
Postoperative stroke (CT-proofed), *n*	52 (5.7%)	42 (6.0%)	10 (4.9%)	0.569
Deep sternal wound infection, *n*	12 (1.3%)	6 (0.9%)	6 (3.0%)	**0.032**
Intensive care unit stay, days	2 (1, 4)	2 (1, 3)	2 (1, 5)	**0.005**
Hospital stay, days	9 (7, 13)	9 (7, 12)	10 (8, 15)	**0.002**
30-day mortality, *n*	36 (4.0%)	23 (3.5%)	13 (7.1%)	**0.031**

The multivariate logistic regression analysis pointed out that older age (> 75 years, OR 2.799; *p* = 0.019), cardiopulmonary bypass time (OR 1.022, *p* < 0.001), cross-clamping time (OR 0.985, *p* = 0.033), and postoperative blood transfusion (OR 4.650, *p* = 0.006) were independent risk factors for mortality (Table 4).

**Table 4:** The multivariate logistic regression analysis for 30 days mortality. Variables included in the regression analysis: age, EuroSCORE II, aortic aneurysm, aortic calcification, COPD, coronary heart disease, chronic renal insufficiency, additional CABG, and cardiopulmonary bypass time

	Odds ratio	CI	*p* value
Age > 75 years	2.799	1.184–6.616	**0.019**
Cardiopulmonary bypass time	1.022	1.013–1.032	**< 0.001**
Cross-clamp time	0.985	0.972–0.999	**0.033**
Postoperative blood transfusion	4.650	1.550–13.952	**0.006**

## Discussion

In the last decades, cerebral tissue protection was the main concern in aortic surgery due to the high susceptibility of the nerve tissue to ischemic adverse effects. As it is known that systemic hypothermia reduces the metabolic tissue rate and consequently protects the brain during cardiac and aortic surgeries, various studies were carried out to investigate the impact of hypothermic circulatory arrest on the neurological outcomes as a result of brain tissue ischemia following aortic surgery [6].

The idea of reduction of cerebral metabolic activity and its oxygen consumption in hypothermic circulatory arrest was confirmed in several studies [7, 8]. According to Yan et al., hypothermia was categorized in four groups according to physiological findings into mild (34–28 °C), moderate (28–20 °C), deep (20–14 °C), and profound (< 14 °C) [9].

A recent study in 2018 from Stewart et al. on the long-term survival and quality of life after hypothermic circulatory arrest in aortic surgery [77% were operated under deep hypothermia (≤ 20 °C), and 23% under moderate hypothermia (20.1 °C–28.0 °C)] showed that patients undergoing surgery of the thoracic aorta achieve a similar long-term life expectancy and health-related quality of life like those of patients undergoing coronary surgery without hypothermic circulatory arrest. These results justify operative treatment in this high-risk patient population. This study supported the idea of using the hypothermic circulatory arrest as a method of cerebral protection in general [10].

However, the ideal level of temperature to be selected during hypothermic circulatory arrest, besides the technique used for cerebral protection, is still controversial. Several studies suggested that prolonged deep HCA more than 20 min reduce the postoperative cognitive function of life in patients undergoing thoracic aortic surgery and was associated with poorer neurological outcomes, necessitating the usage of antegrade (ACP) and retrograde cerebral perfusion (RCP) [2, 11]. On the contrary, other centers do not share the same experience regarding the reduction of cognitive functions with prolonged deep HCA in comparison with ACP and RCP. A review from Ziganshin and coworkers comparing cerebral protection techniques stated that those alternatives failed to induce a better survival and stroke rates than deep HCA. Among a large scale of studies between 2007 and 2012, results of deep HCA were very similar, if not better, when compared to other techniques of cerebral protection [12].

A study from the New York Mount Sinai group showed that HCA (14–40 min) did not have an adverse effect on postoperative cognitive function. On the other side, selective cerebral perfusion (39–83 min) was one of the significant predictors reducing memory and language tests [13].

Chau et al. stated that HCA is a safe and effective method for cerebral protection against ischemic injury during surgeries of the aorta and aortic arch. HCA is equal and, in some other studies, better than ACP and RCP in terms of minimizing stroke and mortality rates as well as in preserving neurocognitive functions. Its simple application provides an optimal field of visualization as well as suitable for emergency cases, which cannot be offered by either ACP or RCP [3]. Moreover, ACP is not always easy to apply and cannot be used in patients with an extended dissected aortic membrane in brachiocephalic or carotid arteries. RCP leads to inadequate neuroprotection due to the inability of a precise estimation of perfusion volume [14, 15].

In our opinion, the tendency to use a higher level of temperature as MHCA more than DHCA to reduce the risk of coagulopathy is increasing nowadays [9, 16, 17]. We considered one of the centers who support this strategy due to the shorter time of rewarming, in order to reduce the duration of surgery and avoid unexpected cognitive adverse outcomes. In 2016, Gong et al. supported our strategy. His study implies the clinical safety and efficacy of MHCA in emergency aortic arch repair, which can provide comparable cerebral and visceral organ protection while decreasing CPB and aortic cross-clamp times without increasing the risk of operative mortality and morbidity [18].

Moreover, a recent study from Kamenskaya et al. aimed to compare the effect of deep HCA (18 °C) vs. moderate HCA (24 °C) combined with antegrade cerebral perfusion (ACP) in cerebral protection during the surgical treatment of chronic dissection of the ascending aorta and aortic arch. The regional hemoglobin oxygen saturation (rSO_2_, %) was compared during surgery. Neurological complications were analyzed during the early postoperative period. Moreover, the study assessed the quality of life (QoL) in the long-term postoperative period (1-year follow-up). The study stated that MHCA with ACP during the surgical treatment of the aorta demonstrated higher qualities of cerebral protection, resulting in reduced neurologic complications during the early postoperative period compared to patients who underwent DHCA during surgery [16].

In comparison with our results, we proved that isolated usage of MHCA without ACP could also achieve satisfactory results regarding the postoperative neurological outcomes in older patients. We agreed with those experiences that recommend the isolated application of MHCA, due to its simplicity and ease of application especially if the time of circulatory arrest is not expected to be longer than the average. Moreover, we figured out the relation between the MHCA and the postoperative neurological outcome in old aged patients.

However, that debate had divided the community of cardiovascular surgeons into various camps, each of them supporting one of these techniques according to their experience and preferences [13, 19, 20].

## Conclusion

Our current analysis reveals that surgical replacement of the ascending aorta using moderate hypothermic circulatory arrest could be applied easily and safely on old aged patients without increasing the risk of neurological complication, even when heavily calcified aorta has to be resected. We encourage the usage of this technique in complexed surgical aortic operation.

### Limitations

The main limitation of this study is its retrospective design. The study is a single-center study and based on a non-randomized analysis of data.

Another limitation is the inability of NIRS to detect other uneventful focal cerebral ischemia, which do not exist in the field of view of the used NIRS.

## Data Availability

The datasets used and/or analyzed during the current study are available from the corresponding author on reasonable request.

## Abbreviations

ACP: Antegrade cerebral perfusion
CABG: Coronary artery bypass grafting
CAM: Confusion assessment method
CT-scan: Computed tomography
MHCA: Moderate hypothermic circulatory arrest
NIRS: Near-infrared spectroscopy
PCP: Postgrade cerebral perfusion
QoL: Quality of life
rSO_2_, %: Oxygen saturation
TIA: Transient ischemic attack
